# A Fluorescent Bioreporter for Acetophenone and 1-Phenylethanol derived from a Specifically Induced Catabolic Operon

**DOI:** 10.3389/fmicb.2015.01561

**Published:** 2016-01-28

**Authors:** Enrico Muhr, Oliver Leicht, Silvia González Sierra, Martin Thanbichler, Johann Heider

**Affiliations:** ^1^Laboratory of Microbial Biochemistry, Department of Microbiology, Philipps-Universität MarburgMarburg, Germany; ^2^Laboratory of Cellular Microbiology, Department of Microbiology, Philipps-Universität MarburgMarburg, Germany; ^3^LOEWE Center for Synthetic MicrobiologyMarburg, Germany; ^4^Max Planck Institute for Terrestrial MicrobiologyMarburg, Germany

**Keywords:** *Aromatoleum aromaticum*, acetophenone, bioreporter, fluorescence microscopy, flow cytometry, immunoblot, mCherry

## Abstract

The β-proteobacterium *Aromatoleum aromaticum* degrades the aromatic ketone acetophenone, a key intermediate of anaerobic ethylbenzene metabolism, either aerobically or anaerobically via a complex ATP-dependent acetophenone carboxylase and a benzoylacetate-CoA ligase. The genes coding for these enzymes (*apcABCDE* and *bal*) are organized in an apparent operon and are expressed in the presence of the substrate acetophenone. To study the conditions under which this operon is expressed in more detail, we constructed a reporter strain by inserting a gene fusion of *apcA*, the first gene of the *apc-bal* operon, with the gene for the fluorescent protein mCherry into the chromosome of *A. aromaticum*. The fusion protein indeed accumulated consistently with the expression pattern of the acetophenone-metabolic enzymes under various growth conditions. After evaluating and quantifying the data by fluorescence microscopy, fluorescence-based flow cytometry and immunoblot analysis, mCherry production was found to be proportional to the applied acetophenone concentrations. The reporter strain allowed quantification of acetophenone within a concentration range of 50 μM (detection limit) to 250 μM after 12 and 24 h. Moreover, production of the Apc-mCherry fusion protein in the reporter strain was highly specific and responded to acetophenone and both enantiomers of 1-phenylethanol, which are easily converted to acetophenone. Other analogous substrates showed either a significantly weaker response or none at all. Therefore, the reporter strain provides a basis for the development of a specific bioreporter system for acetophenone with an application potential reaching from environmental monitoring to petroleum prospecting.

## Introduction

**FIGURE 1:**
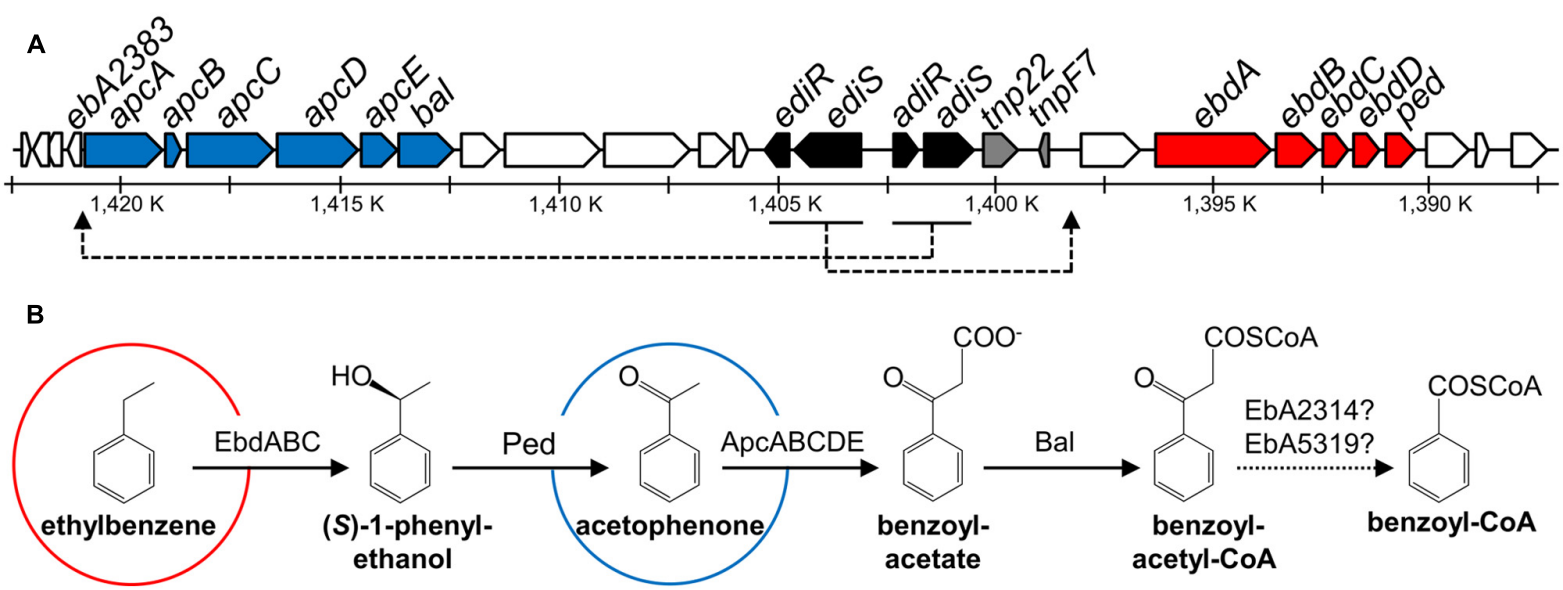
**Scheme of genes and enzymes involved in anaerobic degradation of ethylbenzene and acetophenone in *Aromatoleum aromaticum*.** Modified from Rabus et al. (2002), Kühner et al. (2005), Muhr et al. (2015). **(A)** Predicted functions of gene products are indicated by different colors: blue: acetophenone and ethylbenzene degradation, red: ethylbenzene degradation, black: regulation, gray: transposase/transposase fragment, white: proteins of unknown function. **(B)** Enzyme names: EbdABC: ethylbenzene dehydrogenase, Ped: (*S*)-1-phenylethanol dehydrogenase, ApcABCDE: acetophenone carboxylase, Bal: benzoylacetate CoA-ligase and EbA2314 (accession number CAI07405) or EbA5319 (accession number CAI09148): potential β-ketothiolases. Shown are also genes encoding two predicted two-component regulatory systems, *ediSR* (*tcs2*/*tcr2*) for ethylbenzene degradation induction, and *adiRS* (*tcr1*/*tcs1*) for acetophenone degradation induction.

Among many other aromatic substrates, the β-proteobacterium *Aromatoleum aromaticum* strain EbN1 degrades the aromatic ketone acetophenone (phenylmethylketone) as sole carbon and energy sources (Rabus and Widdel, 1995). When grown anaerobically in the presence of nitrate, it utilizes acetophenone either directly or as a metabolic intermediate in the degradation of ethylbenzene (**Figure 1**), whereas under aerobic conditions, only acetophenone is metabolized, but not ethylbenzene (Rabus and Widdel, 1995; Rabus and Heider, 1998; Heider et al., 1999; Jobst et al., 2010). The industrial use of acetophenone includes applications as solvent and precursor for the production of resins, but also as a food-flavoring agent or fragrance for cosmetics. Acetophenone vapors may cause skin irritation and transient corneal injury in humans after short-term exposure, but no information is available on potential long-term effects (Hagan et al., 1967; US EPA, 1999). Compared to the relatively low toxicity of acetophenone, some of its halogenated derivatives (e.g., the tear gas compound 2′-chloroacetophenone) are highly toxic and cause environmental problems when spilled (Olajos and Salem, 2001). Toxicity of acetophenone toward growth of microorganisms has been reported at concentrations of 0.4–30 mM (Fedorov et al., 1993; Hage et al., 2001; Yang et al., 2008).

Microbial degradation of acetophenone and chloroacetophenones has been known for a long time and was initially believed to be restricted to aerobic microorganisms, which initiate the metabolic pathway by a Baeyer–Villiger oxidation to (chloro)phenyl-acetate esters (Cripps, 1975; Havel and Reineke, 1993). This reaction is catalyzed by a variety of oxygenases that usually accept many different substrate analogs (Jones et al., 1994; Kamerbeek et al., 2003). An oxygen-independent degradation pathway of acetophenone has been discovered in denitrifying bacteria capable of anaerobic ethylbenzene degradation, with *A. aromaticum* EbN1 as the best characterized strain (**Figure 1**; Rabus and Widdel, 1995; Ball et al., 1996; Rabus and Heider, 1998; Champion et al., 1999; Jobst et al., 2010). This pathway starts by oxidizing ethylbenzene to acetophenone in two enzymatic steps via (*S*)-1-phenylethanol, utilizing the molybdoenzyme ethylbenzene dehydrogenase (Johnson et al., 2001; Kniemeyer and Heider, 2001a) and an NAD-dependent (*S*)-1-phenylethanol dehydrogenase (Kniemeyer and Heider, 2001b; Höffken et al., 2006), while acetophenone is further degraded to benzoyl-CoA (Rabus and Heider, 1998; Jobst et al., 2010). *A. aromaticum* also degrades acetophenone as sole substrate, using the same pathway under anaerobic and aerobic growth conditions (Rabus and Widdel, 1995; Ball et al., 1996). The same strain is known to degrade the close chemical analog 4′-hydroxyacetophenone, albeit using a completely different pathway (Wöhlbrand et al., 2008).

Acetophenone degradation by *A. aromaticum* is initiated by a highly complex ATP-dependent acetophenone carboxylase (Apc) which produces benzoylacetate (Jobst et al., 2010). This enzyme has been purified and characterized from cells grown under denitrifying conditions on either ethylbenzene or acetophenone (Jobst et al., 2010). It consists of five subunits (ApcABCDE) that are encoded in an operon together with a gene coding for benzoylacetate CoA-ligase (*bal*; **Figure 1**; Rabus et al., 2002). Four subunits (ApcABCD) form a (αβγδ)_2_ core complex of 485 kDa, which contains tightly bound Zn atoms believed to be essential for carboxylase activity. The fifth subunit (ApcE) is separated from the complex during purification and needs to be added to restore activity *in vitro*. The reconstituted enzyme exhibits a stoichiometry of two ATP hydrolyzed to ADP per acetophenone carboxylated (Jobst et al., 2010). The generated benzoylacetate is subsequently activated to the CoA-thioester by a CoA-ligase (Rabus and Heider, 1998; Rabus et al., 2002; Muhr et al., 2015), and benzoylacetyl-CoA is cleaved by a so far unknown thiolase to acetyl-CoA and benzoyl-CoA, the common intermediate in the anaerobic metabolism of aromatic compounds (Heider and Fuchs, 1997a,b; Carmona et al., 2009; Fuchs et al., 2011). The *apc-bal* operon is part of a larger gene cluster including the *ebd-ped* operon and genes coding for two two-component regulatory systems (**Figure 1**; Rabus et al., 2002).

The enzymes of acetophenone metabolism are apparently regulated in response to the availability of the substrate, but independently of those of ethylbenzene metabolism (Kühner et al., 2005; Rabus et al., 2014; Muhr et al., 2015) or those involved in degradation of the close chemical analogs 4′-ethylphenol and 4′-hydroxyacetophenone (Wöhlbrand et al., 2008; Rabus et al., 2014; Muhr et al., 2015). In order to analyze the mechanisms regulating the acetophenone-metabolic genes, we constructed an *A. aromaticum* strain carrying a chromosomally integrated fusion of the first gene of the *apc-bal* operon (*apcA*) with the gene for the fluorescent protein mCherry (Shaner et al., 2004) and tested for its expression in the presence of various potential inducers. This strain indeed responded to acetophenone and both enantiomers of 1-phenylethanol in a concentration- and time-dependent manner and showed high substrate specificity.

## Materials and Methods

### Bacterial Strains and Growth Conditions

The streptomycin-resistant *A. aromaticum* strain EbN1-SR7 (Wöhlbrand and Rabus, 2009) was grown at 28°C under denitrifying conditions in mineral salt medium on a rotary shaker as described elsewhere (Rabus and Widdel, 1995; Rabus and Heider, 1998; Muhr et al., 2015). Bacterial growth was followed by measuring optical density at 578 nm and the consumption of nitrate by using semiquantitative test strips (Macherey-Nagel, Düren, Germany). For time- and concentration-dependent analysis we inoculated the reporter strain in carbonate-buffered mineral salt media containing 1.5 mM of benzoate in hungate tubes under aerobic conditions. These cultures were incubated for 24 h at 28°C, until they had reached an OD_578_ of 0.4–0.5 and most of the benzoate was consumed (typical residual benzoate concentrations were 0.2 mM). At this point (*t* = 0 h), different concentrations of acetophenone were added. For conjugational plasmid transfer, strain EbN1-SR7 was grown in a modified mineral salt medium described previously (Wöhlbrand and Rabus, 2009).

*Escherichia coli* strains were grown at 37°C in LB media (Sambrook and Russell, 2001). If required, the growth medium was solidified with 1.5% (w/v) agar. Antibiotics were added at the following final concentrations: kanamycin 30–50 μg ml^-1^ and streptomycin 30–50 μg ml^-1^. The diaminopimelate (DAP) auxotrophic *E. coli* strain WM3064, a derivative of strain β2155 (Dehio and Meyer, 1997), was grown on media containing 0.3 mM DAP. *E. coli* strain DH5α was used for all cloning purposes (Taylor et al., 1993).

### DNA Techniques and Plasmid Transfer

All enzymes and kits for the isolation of DNA or the purification of PCR products or restriction fragments were purchased from Thermo Scientific^TM^ (Life Technologies GmbH, Darmstadt, Germany) or Analytik Jena (Jena, Germany). Oligonucleotide primers were ordered from biomers.net GmbH (Ulm, Germany). Sequences were analyzed using pDRAW32 (ACACLONE software) and DNAMAN (Lynnon Biosoft) software.

Plasmids were transferred into *E. coli* strains by transformation using chemically competent cells (Inoue et al., 1990) and into *A. aromaticum* by conjugation (Wöhlbrand and Rabus, 2009). Bacterial mating and conjugational plasmid transfer were performed as described before (Wöhlbrand and Rabus, 2009), with the exception that the DAP-auxotrophic *E. coli* strain WM3064 was used as donor strain.

### Construction of a Chromosomal *apcA-mCherry* Insertion Mutant

**FIGURE 2:**
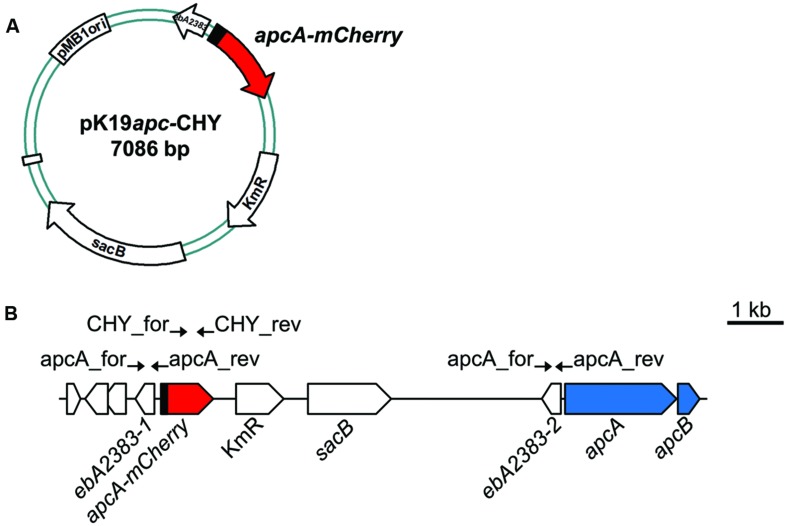
**Construction of *A. aromaticum* strain APC-CHY.** **(A)** Schematic depiction of plasmid pK19*apc*-CHY. **(B)** Reporter strain APC-CHY containing the integrated plasmid pK19*apc*-CHY with a reporter fusion behind the unchanged *apc* upstream sequence resulting in a duplicated copy of the hypothetical gene ebA2383. Primers used for construction are indicated by arrows at their annealing positions.

To generate a strain with a chromosomal integration of an *apcA-mCherry* fusion, we used the suicide-vector pK19mobsacB (Schäfer et al., 1994), which had previously been used to create a knock-out mutant in *A. aromaticum* (Wöhlbrand and Rabus, 2009). The procedure was modified to promote only a single crossover event with the chromosomal DNA, leading to the stable insertion of the entire vector plus the cloned fragment (**Figure 2B**). A fragment of the *mCherry* gene lacking the start codon (starting from third codon) was amplified from plasmid pCHYC-2 (Thanbichler et al., 2007) using primers CHY_for_XbaI (5′-AATCTAGAAAGGGCGAGGAGGATAACATG-3′) and CHY_rev_EcoRI (5′-AAGAATTCTTACTTGTACAGCTCGTCCATG-3′), restricted with *Xba*I and *Eco*RI, and ligated into the equally treated vector pK19mobsacB to generate pK19CHY (6401 bp). In addition, a fragment including 607 bp of the 5′ upstream region and the first 96 bp of the first gene of the *apc*-operon (*apcA*) was amplified from genomic DNA of strain EbN1-SR7 using primers apcA_for_HindIII (5′-GCAAGCTTGGCGATTCACCCGTTTCG-3′) and apcA_rev_XbaI (5′-AATCTAGACGGGGTGGTGTCCACCTTG-3′), restricted with *Hind*III and *Xba*I, and ligated into the equally treated pK19CHY. The resulting plasmid, pK19*apc-*CHY (7086 bp), was sequenced and contained the first 32 codons of *apcA* fused to the *mCherry* gene. It was conjugated into EbN1-SR7, and the transconjugants were purified at least three times on DAP-free media to guarantee the purity of the cultures and absence of residual *E. coli* contamination. The colonies were screened for the presence of plasmid-mediated kanamycin resistance, which should indicate chromosomal integration by a crossover event in the homologous upstream sequence of *apcA*. The proper insertion of the plasmid at the predicted site was verified by colony PCR of the kanamycin-resistant *apcA-mCherry* mutant (data not shown), henceforth designated as strain APC-CHY.

### Fluorescence Microscopy

Cultures of strain APC-CHY grown either aerobically or under denitrifying conditions were exposed to air for at least 1 h at room temperature before being immobilized on a 1% agarose-pad. Cells were analyzed using a Zeiss Axio Observer.Z1 microscope (Zeiss, Germany) equipped with a Zeiss Plan-Apochromat 100×/1.46 numeric-aperture oil differential interference contrast (DIC) M27 objective, a Chroma ET-mCherry filter set, and a pco.edge sCMOS camera (PCO, Germany). Images were analyzed and processed with ImageJ 1.48v (Wayne Rasband, National Institutes of Health, USA), Metamorph 7.7.5 (Universal Imaging Group, USA), and Illustrator CS5 (Adobe Systems, USA). All fluorescence images (500 ms exposure) shown in this study were scaled equally (monochrome, threshold setting 250/10,000) for comparison in brightness. For the quantitative analysis of relative mean and maximum fluorescence signal response (threshold set at 750 units), the fluorescence images were divided into 10–15 sectors each containing about 25 cells. Each sector was analyzed separately, and average values were generated.

### Flow Cytometry

For fluorescence–based flow cytometry, bacterial cells were washed two times with fresh mineral salt medium in which the substrates were omitted and diluted 1:100 in tethering buffer (10 mM K_2_HPO_4_, 10 mM KH_2_PO_4_, 10 mM lactic acid, 0.1 mM EDTA, and 1 μM L-methionine, pH 7.0). Fluorescence-activated cell analysis was carried out on a BD LSRFortessa^TM^ SORP flow-cytometer (BD Biosciences, Franklin Lakes, NJ, USA; Piatkevich and Verkhusha, 2011; Telford et al., 2012). Fluorescence was detected using a 561-nm laser (yellow–green) at 100 mW for excitation and a 610/20 bandpass filter. The forward and side scatter values were monitored using a 488-nm laser (blue) at 50 mW. The acquired data were analyzed using BD FACSDiva^TM^ software version 8.0 (BD Biosciences, Franklin Lakes, NJ, USA) with data collected in FCS 3.0 file format.

### Electrophoretic Methods and Immunoblotting

Defined amounts of protein (50 μg) from crude extracts were separated by discontinuous sodium dodecyl sulfate-polyacrylamide gel electrophoresis (SDS-PAGE) using 12.5% (w/v) polyacrylamide gels in the Mini-PROTEAN Tetra System (Bio-Rad, Munich, Germany) as described elsewhere (Laemmli, 1970). For extract preparation, cell suspensions were disrupted by sonication and cell debris and membranes were removed by ultracentrifugation at 100,000 × *g* and 4°C for 1 h. Protein concentrations were determined using a Coomassie dye binding assay with bovine serum albumin as a standard (Bradford, 1976). Immunodetection with anti-mCherry antiserum was performed as described before (Jung et al., 2014). For quantitation of the immunocomplexes, the signals were recorded by a ChemiDoc MP imaging system as volume (intensity) of the respective bands using Image Lab 5.0 software (Bio-Rad, Munich, Germany). All ApcA-mCherry fusion protein signals are presented in arbitrary units (a.u.), where one unit corresponds to an original volume (intensity) readout value of 10^6^. Data were fitted using GraphPad Prism 4 (GraphPad Software, San Diego, CA, USA). To control for equal loading, all membranes were stained with Ponceau S [0.1% (w/v) in 5% (v/v) acetic acid; Sigma-Aldrich, St. Louis, MO, USA].

### Determination of Benzoate Concentrations

To determine the concentrations of benzoate in culture supernatants, 10% (v/v) of a 1 M NaHSO_4_ solution was added to reach a final pH of ∼2.0. After centrifugation (15,000 × *g* for 5 min), the absorbance at 230 nm was detected using UV-cuvettes (Robinson, 1991) and the concentration of benzoic acid was calculated from its extinction coefficient (ε = 11,900 M^-1^ cm^-1^).

## Results

### Construction of a Chromosomal Fluorescent Reporter Fusion

Previous studies addressing enzyme activities in *A. aromaticum* cells grown on various substrates indicated that acetophenone-catabolic genes are exclusively induced in cells grown on acetophenone or ethylbenzene (Jobst et al., 2010; Rabus et al., 2014; Muhr et al., 2015). In order to further elucidate the induction pattern of these genes *in vivo*, we constructed a translational fusion of the first 32 codons of the *apcA* gene with the *mCherry* gene to generate a fluorescent reporter system (**Figure 2**). To avoid potential problems resulting from multiple copies of a plasmid-borne reporter gene, we integrated the *apcA-mCherry* reporter fusion into the chromosome of strain EbN1-SR7 (Wöhlbrand and Rabus, 2009) by inserting plasmid pK19*apc*-CHY upstream of the first gene of the *apc-bal* operon through a single cross-over event (**Figure 2**). The resulting mutant strain, APC-CHY, carried the reporter fusion behind the unchanged *apc* upstream sequence plus a duplicated copy of 607 bp of the *apc* upstream sequence, followed by the entire *apc-bal* operon. The identity of this strain was verified by its kanamycin resistance and by testing for proper integration of the plasmid using PCR analysis. Additionally, its growth properties with ethylbenzene, acetophenone, or benzoate did not differ significantly from those of the wild-type strain (data not shown).

### Reporter Gene Response to Different Growth Conditions

**FIGURE 3:**
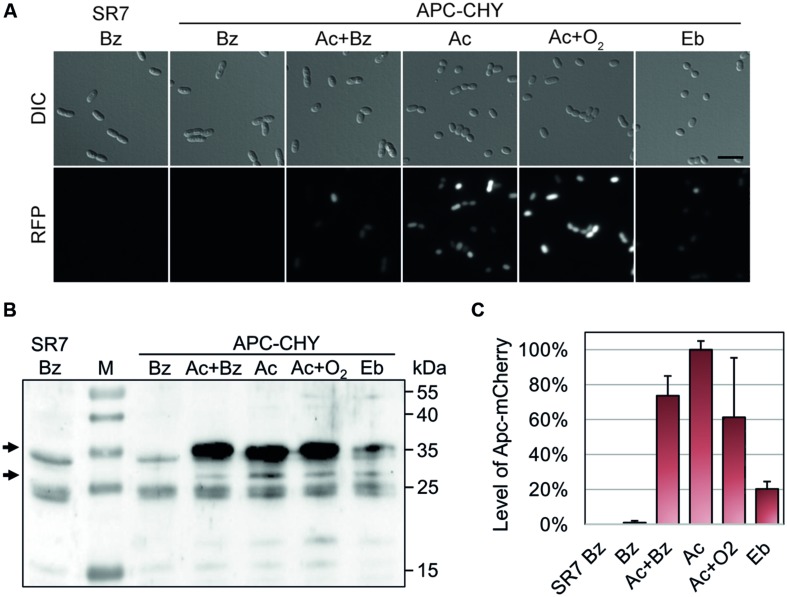
**Qualitative and quantitative analysis of ApcA-mCherry fusion protein production in strain APC-CHY.** Cultures were grown to mid-exponential phase (72 h) under denitrifying conditions on 4 mM benzoate (Bz), 2 mM acetophenone plus 2 mM benzoate (Ac+Bz), 2 mM acetophenone (Ac), or 2 mM ethylbenzene (Eb). In addition, one culture was grown on 2 mM acetophenone under aerobic conditions (Ac+O_2_). The parental strain EbN1-SR7 grown on benzoate served as control (SR7 Bz). **(A)** Differential interference contrast (DIC) and fluorescence microscopy images (RFP, 500 ms) of the cells. Scale bar, 5 μm. All fluorescence microscopy images were scaled equally (250/10,000) and shown in monochrome for comparison of differences in brightness. **(B)** Immunoblot analysis of different cell free extracts (50 μg) of APC-CHY using anti-mCherry antiserum. Molecular masses of the protein standard (M) are given along the right margin. Arrows indicate the positions of the ApcA-mCherry fusion product (30.1 kDa) and a degradation product corresponding in size to free mCherry (∼27 kDa). **(C)** Quantification of signal intensities obtained for the ApcA-mCherry by immunoblot analysis in relative units (%). The values given are the average of at least three parallel measurements. Error bars indicate the standard deviation.

The reporter strain derived from *A. aromaticum* was grown to mid-exponential phase (72 h) under different conditions in carbonate-buffered mineral salt media. The expression of the reporter gene fusion was observed in parallel by fluorescence microscopy and quantitative immunoblotting with antiserum against mCherry (**Figure 3**). We observed increased fluorescence intensities in all APC-CHY cells grown anaerobically on 2 mM acetophenone or ethylbenzene as well as in those grown aerobically on acetophenone, whereas none of the cultures grown on 4 mM benzoate, 1 mM toluene, or 1 mM 4′-ethylphenol showed significant fluorescence intensities (**Figure 3A**; cultures on toluene or 4′-ethylphenol not shown).

The presence of ApcA-mCherry was additionally confirmed by quantitative immunoblotting of extracts separated on an SDS gel (**Figure 3B**). The antiserum reacted strongly with ApcA-mCherry (30.1 kDa) and with a protein corresponding in size to free mCherry protein (∼27 kDa), which likely results from degradation of the fusion protein in cells grown on acetophenone (**Figures 3B,C**). The strongest signal intensity of ApcA-mCherry in cells grown on acetophenone was set as 100%. In comparison, cells grown anaerobically on benzoate plus acetophenone or aerobically on acetophenone showed signal intensities of 75 and 60%, respectively. Cultures of strain APC-CHY grown on ethylbenzene showed signal intensities of 20% under the same conditions. In contrast, controls grown anaerobically on benzoate did not show significant immunoblot signals (**Figures 3B,C**). The few cross-reacting bands in the control show the same pattern as an immunoblot of the parental strain without chromosomally integrated mCherry and are therefore unrelated to the mCherry protein (**Figure 3B**).

### Development of a Quantitative Acetophenone Sensor System

We examined whether the newly constructed reporter system can be used to quantify the concentrations of acetophenone in growth media of *A. aromaticum*. To avoid problems with the oxygen demand for developing the mCherry fluorescence (Shaner et al., 2004), we decided to shift to aerobic growth conditions for setting up a sensory system. To this end, we inoculated the reporter strain in carbonate-buffered mineral salt media containing 1.5 mM of benzoate. After 24 h (*t* = 0 h), different concentrations of acetophenone were added and samples were taken for up to 96 h. The respective reporter output values were analyzed by quantitation of the relative fluorescence intensities from fluorescence microscopy images as well as fluorescence based flow cytometry and confirmed by quantitative immunoblot analysis of ApcA-mCherry signals.

**FIGURE 4:**
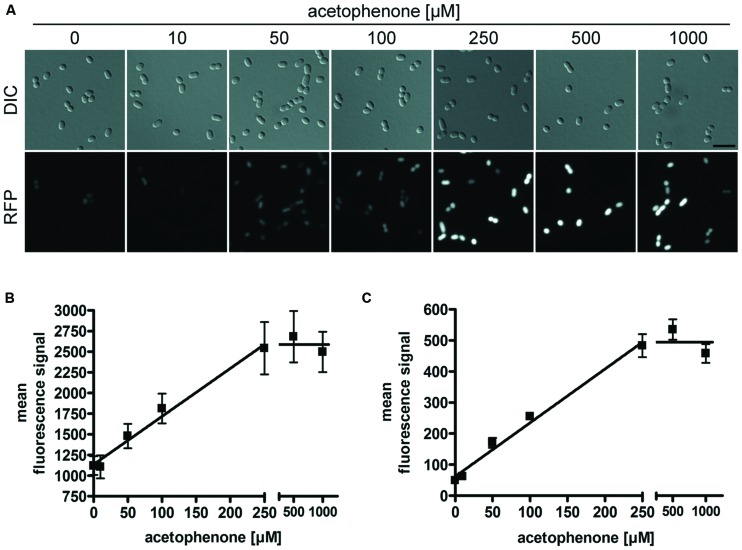
**Acetophenone-dependent fluorescence output.** Cultures of strain APC-CHY were measured for mCherry fluorescence after 24 h of exposure to different concentrations of acetophenone. **(A)** DIC and fluorescence microscopy images for different acetophenone concentrations were shown in monochrome and scaled equally for comparison of differences in brightness. Scale bar, 5 μm. **(B)** Quantitation of relative mean fluorescence signals from fluorescence microscopy images. The values given are the average of the values obtained from at least 50 parallel measurements. **(C)** Relative mean fluorescence signals determined by flow cytometric analysis of 10,000 cells per culture from two biological replicates per acetophenone concentration. **(B,C)** Error bars indicate the standard deviations. Data were fitted by linear regression between 0 and 250 μM acetophenone, yielding *R*^2^ values of 0.984 **(B)** and 0.988 **(C)**. The fitted lines correspond to slopes of 5.81 μM^-1^ **(B)** and 1.73 μM^-1^ **(C)** and background values of 1136 units **(B)** and 61.8 units **(C)**.

Various tested cultures showed a strict dependence of their fluorescence output on the applied acetophenone concentrations. To illustrate this correlation, samples of two biological replicates were taken 24 h after different concentrations of acetophenone (0–1000 μM) had been added. From each replicate at least two fluorescence microscopy images were taken and scaled equally for comparison of differences in brightness (**Figure 4A**). The acetophenone-dependent relative fluorescence signals were quantified by measuring the mean and maximum fluorescence signals of individual cells (threshold set at 750 units) and averaging the data. In both cases, a linear correlation was observed between acetophenone concentration and fluorescence output up to an acetophenone concentration of 250 μM (**Figure 4B**, **Supplementary Figure S1**). For both evaluation methods, the fluorescence output was saturated at acetophenone concentrations over 250 μM. Therefore, the reporter strain allows quantification of acetophenone within a concentration range of 50 μM (detection limit) to 250 μM.

The correlation of acetophenone concentration with mCherry fluorescence was also independently proven by flow cytometric analysis of samples taken from the same cultures that had been used for fluorescence microscopic analysis. To achieve this, 20,000 cells per acetophenone concentration from each replicate were analyzed by measuring their relative mean fluorescence signals (**Figure 4C**). Again, a linear correlation was observed for concentrations up to 250 μM acetophenone, which turned over to a saturated reaction at higher substrate concentrations, exactly mimicking the results obtained from fluorescence measurements using microscopy images.

### Time-Dependence of the Assay

**FIGURE 5:**
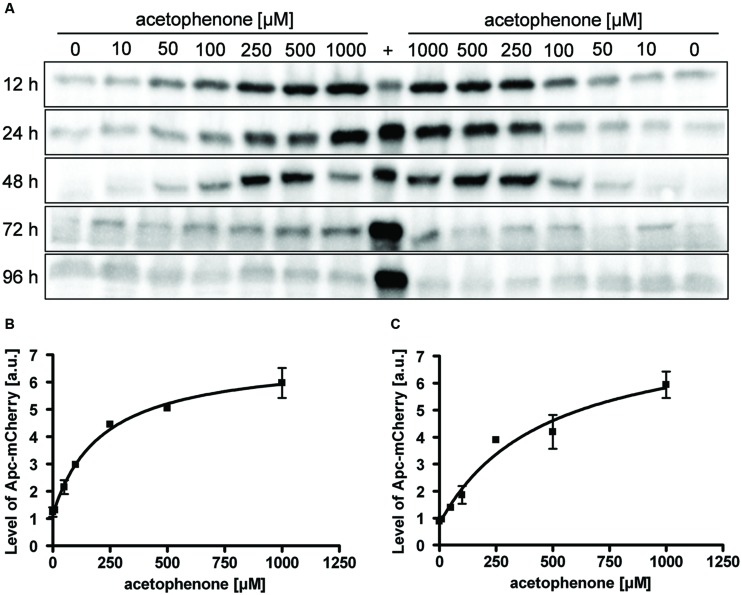
**Time- and concentration-dependency of reporter protein content.** **(A)** Cultures of strain APC-CHY were tested for their content of ApcA-mCherry fusion protein by immunoblot analysis after various times of exposure to different concentrations of acetophenone. Samples were taken after the indicated time points given along the left margin. The lanes were loaded with cell free extracts (50 μg) from two biological replicates per acetophenone concentration and probed with anti-mCherry antiserum. A control culture of strain APC-CHY grown under denitrifying conditions on 2 mM acetophenone to mid-exponential phase (72 h, see **Figure 3B**) served as control (labeled with +). **(B,C)** Quantification of ApcA-mCherry signal intensities from the immunoblot analysis after 12 h **(B)** and **(C)** 24 h. The values given are the average of at least three parallel measurements including standard deviations in arbitrary units (a.u.). Data were fitted by the saturation kinetics equation given above, *R*^2^ values were 0.996 **(B)** or 0.976 **(C)**. Curve parameters: max = 5.812 a.u. **(B)** or 7.395 a.u. **(C)**, *K* = 213.7 μM **(B)** or 467.4 μM **(C)**, and background values 1.133 a.u. **(B)** or 0.786 a.u. **(C)**. It should be noted that the units in **(B,C)** are not directly comparable because the experiments were performed independently.

In addition to the concentration dependence, the quantitation of various samples taken after different incubation times showed a time-dependency of the fluorescence output. Apart from using the two fluorescence-based methods mentioned above, we analyzed the same samples for presence of ApcA-mCherry fusion proteins directly, using quantitative immunoblot analysis of the respective cell free extracts with antiserum against mCherry. Samples were analyzed for up to 96 h after exposing the cultures to different concentrations of acetophenone (**Figure 5A**). In contrast to the linear correlation between substrate concentration and output observed for the fluorescence-based methods, the direct quantitation of fusion protein yielded obvious saturation kinetics curves, at least for the samples analyzed after 12 and 24 h (**Figures 5B,C**). The data fitted very well to the following equation, where *P* is the measured immunoblot signal, *c* is the acetophenone concentration, *max* is the saturation value, *K* is the concentration at half-maximum saturation, and *B* is the background value without added acetophenone.

$$P=\frac{\max*c}{K+c}+B$$

As an additional control, a sample of strain APC-CHY grown under denitrifying conditions on 2 mM acetophenone to mid-exponential phase (72 h, see **Figure 3B**) was included in the immunoblots (**Figure 5A**, labeled with +). The concentration dependence of the Apc-mCherry levels after 12 and 24 h was very similar and again allowed a good resolution of acetophenone concentrations in the range of 50–250 μM. However, as seen in **Figure 5A**, the correlation between acetophenone concentration and Apc-mCherry content deteriorated with longer exposure times. Similar results were obtained by fluorescence microscopy from APC-CHY cells after 48 h exposure as well as by flow cytometry analysis after 48 and 72 h exposure (data not shown).

### Specificity of Acetophenone Sensing

**FIGURE 6:**
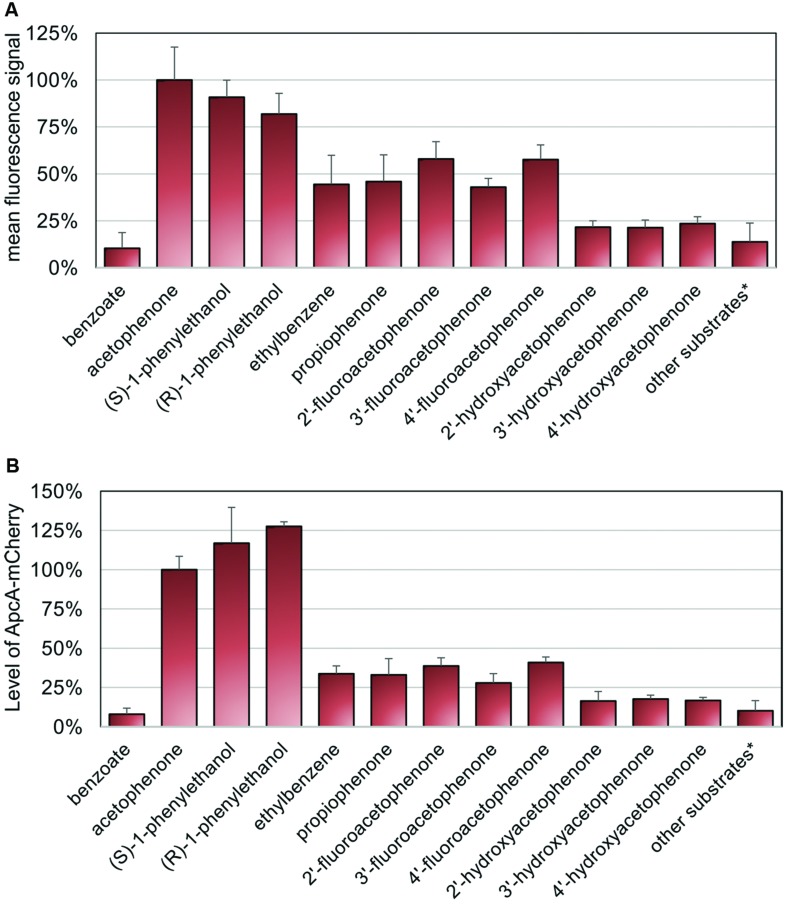
**Specificity of sensing.** Cultures of strain APC-CHY were analyzed for mCherry fluorescence after 16 h of exposure to different substrates at final concentrations of 0.5 mM. Substrates yielding only background fluorescence, whose recorded values were treated summarily: 2-phenylethanol, phenylacetaldehyde, 2′-chloroacetophenone, 2′-methylacetophenone, 4′-methylacetophenone, styrene oxide, styrene, toluene, 4-cresol, phenol, and 4′-ethylphenol. **(A)** Quantitation of relative mean fluorescence signals from fluorescence microscopy images. The values given are the average of the data obtained for at least 30 parallel measurements. **(B)** Quantification of signal intensities of ApcA-mCherry fusion proteins from immunoblot analysis of cell free extracts (50 μg) using anti-mCherry antiserum. The values given represent the average of at least three parallel measurements. **(A,B)** Error bars indicate the standard deviations in relative units (%).

Activity of acetophenone carboxylase (ApcABCDE) has been shown to be highly specific toward its natural substrate acetophenone (Jobst et al., 2010; Muhr et al., 2015). Therefore, we investigated the fluorescence signal response to metabolic precursors as well as different chemical analogs of acetophenone using the same experimental procedure described above. All these substrates were added individually to a culture pre-grown on benzoate for 24 h under aerobic conditions at final concentrations of 0.5 mM. After 16 h of exposure, samples were analyzed by fluorescence microscopy (**Supplementary Figure S2**). The relative mean fluorescence signals (threshold set at 750 units) as well as the maximum fluorescence signals were quantified from fluorescence microscopy images as described above (**Figure 6A**, **Supplementary Figure S3**). The highest induction levels were observed if acetophenone or its immediate metabolic precursors, (*S*)- or (*R*)-1-phenylethanol were added (**Figure 6A**). The response obtained with acetophenone was taken as 100% reference. In both evaluation variants, the (*S*)-enantiomer of 1-phenylethanol showed a slightly higher fluorescence signal response than the (*R*)-enantiomer, reaching a mean value of 91% and a maximum value of 94% of the level obtained with acetophenone, compared to values of 82 and 80%, respectively (**Figure 6A**, **Supplementary Figure S3**). The inducing effect of the alcohols is most probably due to their conversion to acetophenone, as suggested by analyzing the concentration dependence for (*S*)-1-phenylethanol, which revealed virtually identical saturation kinetics as determined for acetophenone (**Supplementary Figure S4**).

Interestingly, the fluorescence signals observed under aerobic growth conditions with added ethylbenzene had approximately the same intensities as the ones obtaind for the close acetophenone analog propiophenone with mean values of 44 and 46% of the reference value, respectively (**Figure 6A**). Among all other substrate analogs tested, only the fluoroacetophenone isomers generated relatively high mean fluorescence signal values of 58, 43 and 58%, whereas cultures induced with the hydroxyacetophenone isomers showed very weak signals of 21–24% of the reference. A number of other tested compounds yielded only fluorescence values similar to the level of the negative control with benzoate (**Figure 6A**, **Supplementary Figure S2**). These substrates included further ring-substituted acetophenone derivatives such as 2′-chloroacetophenone, 2′-methylacetophenone, and 4′-methylacetophenone, the hydrocarbon analogs toluene and styrene, the phenolic analogs phenol, 4-cresol and 4′-ethylphenol, as well as the side chain analogs 2-phenylethanol, phenylacetaldehyde, and styrene oxide (for recorded data see **Supplementary Figure S2**).

The relative fluorescence intensities determined for the various substrates were confirmed by quantitative immunoblot analysis (**Figure 6B**, **Supplementary Figure S5**). The signal intensities of ApcA-mCherry proteins were quantified and compared to cells induced with 0.5 mM acetophenone (set as 100%). As for the data evaluated by fluorescence microscopy, the highest content of ApcA-mCherry fusion protein was observed if acetophenone or its immediate metabolic precursors (*S*)- or (*R*)-1-phenylethanol were present. The induction levels measured for the (*S*)- and (*R*)-enantiomers of the alcohol were even 1.2 and 1.3 times higher than with acetophenone, respectively (**Figure 6B**). In contrast, control cells grown on benzoate showed only a basal level of 7.9% of the value obtained with acetophenone. Moreover, as observed by fluorescence microscopy, cells exposed to the fluoroacetophenone isomers showed a medium content of ApcA-mCherry fusion protein (39, 28, and 41%, respectively, compared to acetophenone), whereas the hydroxyacetophenone isomers yielded very weak induction levels in the range of 16–18% of the reference value. All other substrates tested in this study gave rise to Apc-mCherry levels of less than 16%, which were very close to the level of the control.

## Discussion

In this study we present the first fluorescent reporter gene fusion constructed in the denitrifying betaproteobacterium *A. aromaticum*, which is a highly versatile degrader of many aromatic compounds (Rabus and Widdel, 1995; Rabus and Heider, 1998; Boll et al., 2002; Rabus et al., 2005, 2014). A strain containing a chromosomally integrated fusion of the first gene of the acetophenone-metabolic *apc-bal* operon with the gene for the fluorescent mCherry protein appears to serve as a reliable reporter system for the presence of certain aromatic compounds without changing the physiological properties of the host strain. Like most other available fluorescent protein reporter systems, mCherry is dependent on exposure to oxygen for the maturation of the protein-derived fluorophore, but it exhibits a relatively fast maturation rate (Shaner et al., 2004, 2005; Merzlyak et al., 2007). We observed that even for cells from strictly anaerobic cultures, exposure of the samples to air for 60 min (including the required processing time for microscopy or other analytical procedures) was sufficient to ensure maturation of the mCherry fusion proteins. Cultures grown under aerobic conditions showed full fluorescence intensities already without preincubation. The presence and quantity of the mCherry fusion proteins was evaluated by three independent methods and showed an excellent correlation between reporter gene expression and the growth conditions under which the enzymes encoded by the induced operon are active (Rabus et al., 2002, 2014; Kühner et al., 2005; Muhr et al., 2015). It was also confirmed here that the *apc-bal* operon is highly induced during acetophenone metabolism under both anaerobic and aerobic growth conditions.

The reporter strain constructed in this study was used to establish a quantitative signal/response relation for the physiological substrate acetophenone based on three independent detection systems. Fluorescence intensities obtained from fluorescence microscopy images or flow cytometric analysis as well as mCherry fusion protein contents were proportional to the applied acetophenone concentrations, providing an effective means to determine unknown concentrations over a range spanning almost one order of magnitude. We observed a linear dependence of the fluorescence signals with acetophenone concentrations of up to 250 μM for the two fluorescence-based detection methods, which turned abruptly into saturation at higher substrate concentrations. In contrast, the concentration dependence of immunologically detected fusion protein was best described by saturation kinetics. This apparent discrepancy might be due to self-absorbance phenomena at high concentrations of fluorescent proteins in the cytoplasm. The readout of the system was very similar after 12 and 24 h of incubation and only deteriorated after 48 h or longer incubation times, probably because of different extents of degradation of the added acetophenone. Another explanation could lie in toxic or growth limiting effects of acetophenone when added at high concentrations of 0.5 and 1 mM.

Notably, the sensing system seems to be rather specific for acetophenone and its immediate metabolic precursors, (*S*)- or (*R*)-1-phenylethanol. Its inability to discriminate between the 1-phenylethanol enantiomers and acetophenone was expected, since *A. aromaticum* is known to contain several alcohol dehydrogenases with significant activities capable of interconverting acetophenone and the 1-phenylethanol enantiomers (Kniemeyer and Heider, 2001b; Rabus et al., 2005; Höffken et al., 2006). This notion is corroborated by the identical response kinetics recorded for (*S*)-1-phenylethanol and acetophenone. Weaker, but still significant fluorescence signals were also recorded after exposure to ethylbenzene, propiophenone, and the isomers of fluoroacetophenone. This suggests that lack of the oxo-group of acetophenone, elongation of the side chain by one C, or presence of a fluoro-substituent at the aromatic ring still allow partial recognition by the regulator protein(s) responsible. Remarkably, propiophenone and the fluoroacetophenones are also among the few substrates efficiently carboxylated by acetophenone carboxylase (Jobst et al., 2010).

In contrast to either of these compounds, the hydroxyacetophenone isomers elicit almost no response, while other ring-substituted analogs like 2′-chloroacetophenone, 2′- or 4′-methylacetophenone only yield values in the range of the negative control. This correlates well with the respective bond lengths, which are shortest for fluoro- and subsequently longer for hydroxyl-, methyl-, and chloro-substituents at aromatic rings (Clugston and Flemming, 2000). Therefore, the fluoroacetophenones are the closest mimics of acetophenone, whereas larger substituents prevent the respective molecules from binding to either the catalytic enzyme or the respective regulator(s). The absence of any response with 2-phenylethanol, styrene oxide, or phenylacetaldehyde indicates the regiospecifity of recognition, which probably requires the correct placement of the oxo group. Moreover, the lack of induction by toluene or styrene compared to a weak inducing effect by ethylbenzene points to the importance of proper size and orientation of the side chain of an inducing compound.

Remarkably, some of the weakly inducing compounds (propiophenone or the fluoroacetophenone isomers) do not serve as growth substrates for *A. aromaticum*, whereas the (almost) non-inducing 4′-hydroxyacetophenone is degraded by a completely different and specifically induced pathway (Wöhlbrand et al., 2008; Rabus et al., 2014; Muhr et al., 2015). It seems that an efficient regulatory machinery has evolved in *A. aromaticum* to specifically discriminate between inducing the enzymes of acetophenone and 4′-hydroxyacetophenone degradation. Therefore, the reporter strain can principally be used as a rather specific bioreporter system for the presence of acetophenone (plus some closely related chemical analogs) in a concentration range of 50–250 μM. Because acetophenone as well as the 1-phenylethanol isomers are common intermediates of aerobic and anaerobic pathways of ethylbenzene degradation (Filipovic et al., 1992; Bernhardt, 2006; Carmona et al., 2009; Fuchs et al., 2011; Rabus et al., 2014), a specific detection system for these compounds may be useful for applications in environmental monitoring or even in prospecting for new petroleum reservoirs, based on indirectly screening for hydrocarbons via their degradation products. Alternative detection systems for acetophenone include GC/MS methods which reach detections limits of around 80 nM (EPA method index: EPA-OSW 8270D). However, we think that a fluorescence-based method which discriminates the target substrates in complex mixtures of other compounds may be preferable for a fast field survey of environmental samples. Typical acetophenone concentrations in pristine environments are far below the detection limit of the bioreporter system (<10 nM), but they are expected to rise above the detection limit in oil-contaminated sites, judging from ethylbenzene contents of crude oil of up to 40 mM (Kolpin et al., 2002).

It seems to be a general property of anaerobic aromatics-degrading bacteria that they use more substrate-specific catabolic pathways than their aerobic counterparts (Bernhardt, 2006; Fuchs et al., 2011), which may go along with higher substrate specificities of the corresponding regulatory systems. These regulatory systems of anaerobic aromatics degraders (compared to mostly unspecific systems in aerobic bacteria) may provide a basis for the development of many more bioreporter systems. The suicide-vector based system for constructing specific sensor strains for aromatic compounds presented in this study can easily be adapted more broadly to other specifically regulated catabolic genes of *A. aromaticum* or other bacteria for detecting and quantifying these compounds.

## Author Contributions

EM carried out the practical work, OL took and analyzed the fluorescence microscopy images, SGS performed the flow cytometry analysis, MT provided advice on fluorescence microscopy experiments and proofread the manuscript. EM and JH designed the experiments, analyzed the results, and wrote the manuscript.

## Conflict of Interest Statement

The authors declare that the research was conducted in the absence of any commercial or financial relationships that could be construed as a potential conflict of interest.
