# Supplementary material for: A Fluorescent Bioreporter for Acetophenone and 1-Phenylethanol derived from a Specifically Induced Catabolic Operon
Source: Front Microbiol. 2016 Jan 28;6:1561. doi: 10.3389/fmicb.2015.01561 (PMC4729919; doi:10.3389/fmicb.2015.01561)
Supplement: Supplementary file 1 [file Data_Sheet_1.DOCX]

Supplementary Material

A fluorescent bioreporter for acetophenone and 1-phenylethanol derived from a specifically induced catabolic operon

**Enrico Muhr, Oliver Leicht, Silvia González Sierra, Martin Thanbichler, Johann Heider***

*** Correspondence:** Johann Heider, Laboratory of Microbial Biochemistry, Department of Microbiology, Philipps Universität Marburg, Karl-von-Frisch-Str. 8, 35043 Marburg, Germany.
[heider@biologie.uni-marburg.de](mailto:heider@biologie.uni-marburg.de)

# Figure Legends

**Figure S1. Quantitation of maximum fluorescence signals for acetophenone-dependent fluorescence output from fluorescence microscopy images.** For images see Fig. 4A. The values given are the average of the values obtained from at least 50 parallel measurements. Error bars indicate the standard deviations. Data were fitted by linear regression between 0 and 250 µM acetophenone, yielding R² values of 0.997, a slope of 44.63 µM^-1^, and a background value of 2303 units.

**Figure S2. Specificity of sensing acetophenone analogues.** DIC and fluorescence microscopy images (RFP, 500 ms) from cultures of strain APC-CHY after 16 h of exposure to different substrates at final concentrations of 0.5 mM (see Fig. 6): #1 benzoate, #2 acetophenone, #3 ethylbenzene, #4 propiophenone, #5 (*S*)-1-phenylethanol, #6 (*R*)-1-phenylethanol, #7 2-phenylethanol, #8 phenylacetaldehyde, #9 2'-hydroxyacetophenone, #10 3'-hydroxyacetophenone, #11 4'-hydroxyacetophenone, #12 2'-fluoroacetophenone, #13 3'-fluoroacetophenone, #14 4'-fluoroacetophenone, #15 2’-chloroacetophenone, #16 2’-methylacetophenone, #17 4’-methylacetophenone, #18 styrene oxide, #19 styrene, #20 toluene, #21 4-cresol, #22 phenol, #23 4’-ethylphenol. All fluorescence microscopy images were scaled equally (250/10,000) and shown in monochrome for comparison of differences in brightness. Scale bar, 5 µm. Each picture shows a representative detail view of the full size images.

**Figure S3. Quantitation of maximum fluorescence signals with acetophenone analogues from fluorescence microscopy images.** For images see Fig. S3. The values given are the average of the data obtained for at least 30 parallel measurements. Error bars indicate the standard deviations in relative units (%).

**Figure S4. Concentration-dependency of reporter protein content to (*S*)-1-phenylethanol. (A)** Cultures of strain APC-CHY were tested for their content of ApcA-mCherry fusion protein by immunoblot analysis after 12 h exposure to different concentrations of (*S*)-1-phenylethanol. (**B**) Quantification of ApcA-mCherry signal intensities from the immunoblot analysis. The values given are the average of at least three parallel measurements including standard deviations in arbitrary units (a.u.). Data were fitted by the saturation kinetics equation given in this study, with an R² value of 0.974. Curve parameters: max = 9.041 a.u., K = 168.9 µM, background value = 0.186 a.u.. It should be noted that the units are not directly comparable to Fig. 5 because the experiments were performed independently.

**Figure S5. Immunoblot analysis of sensing specificity.** Cultures of strain APC-CHY were analyzed for their content of ApcA-mCherry fusion protein by immunoblot analysis of cell free extracts (50 µg) using anti-mCherry antiserum after 16 h of exposure to different substrates at final concentrations of 0.5 mM (see Fig. 6): #1 benzoate, #2 acetophenone, #3 ethylbenzene, #4 propiophenone, #5 (*S*)-1-phenylethanol, #6 (*R*)-1-phenylethanol, #7 2-phenylethanol, #8 phenylacetaldehyde, #9 2'-hydroxyacetophenone, #10 3'-hydroxyacetophenone, #11 4'-hydroxyacetophenone, #12 2'-fluoroacetophenone, #13 3'-fluoroacetophenone, #14 4'-fluoroacetophenone, #15 2’-chloroacetophenone, #16 2’-methylacetophenone, #17 4’-methylacetophenone, #18 styrene oxide, #19 styrene, #20 toluene, #21 4-cresol, #22 phenol, #23 4’-ethylphenol. Molecular masses of the protein standard (M) are given along the right margin. Arrows indicate the positions of the ApcA-mCherry fusion product (30.1 kDa) and a degradation product corresponding in size to free mCherry (~27 kDa). It should be noted that the immunoblot analysis are not directly comparable because the experiments were performed independently.
