# Supplementary figures and images for: A Fluorescent Bioreporter for Acetophenone and 1-Phenylethanol derived from a Specifically Induced Catabolic Operon
Source: Front Microbiol. 2016 Jan 28;6:1561. doi: 10.3389/fmicb.2015.01561 (PMC4729919; doi:10.3389/fmicb.2015.01561)

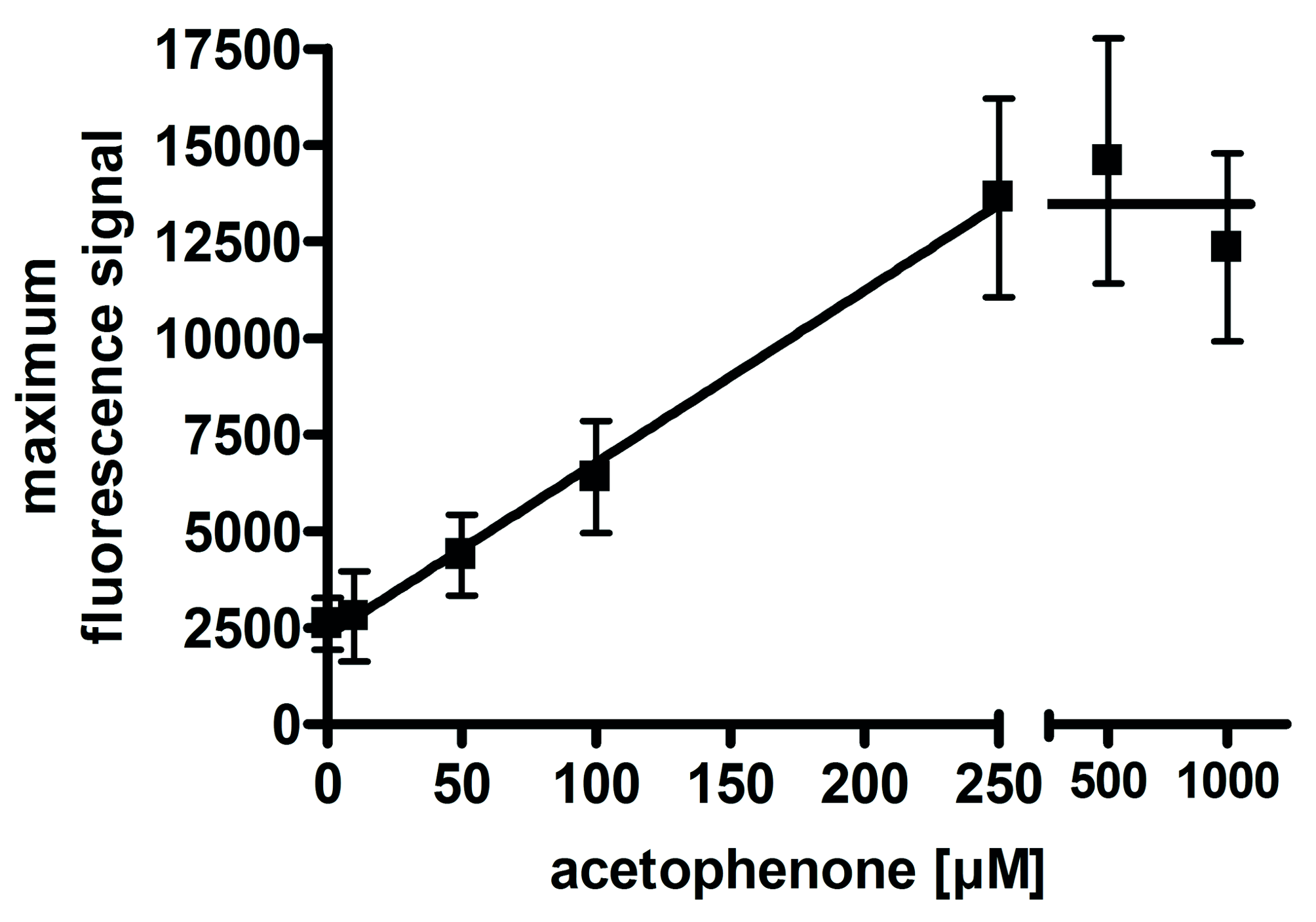

Supplement: Supplementary file 2 [file Image_1.TIF]

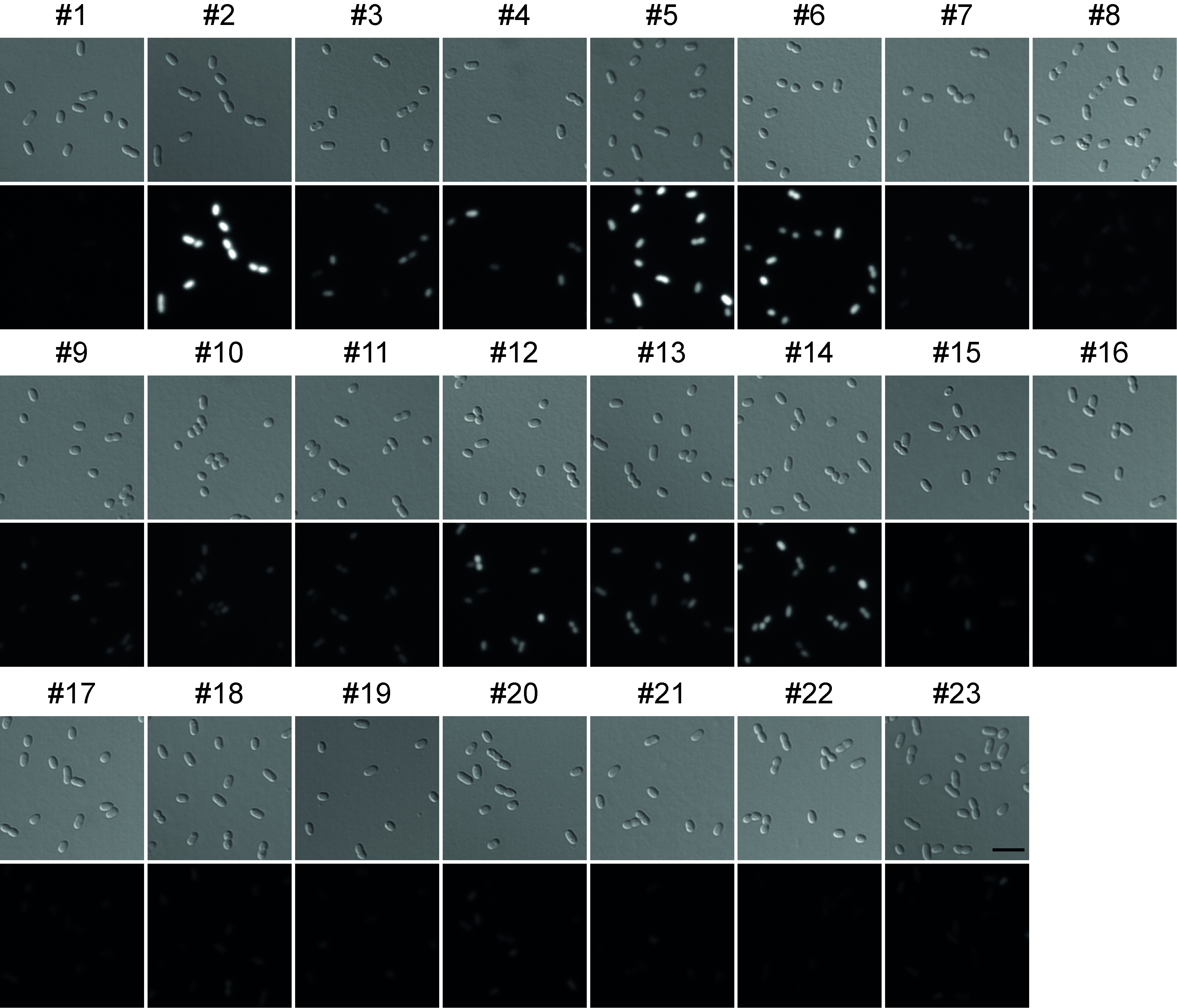

Supplement: Supplementary file 3 [file Image_2.TIF]

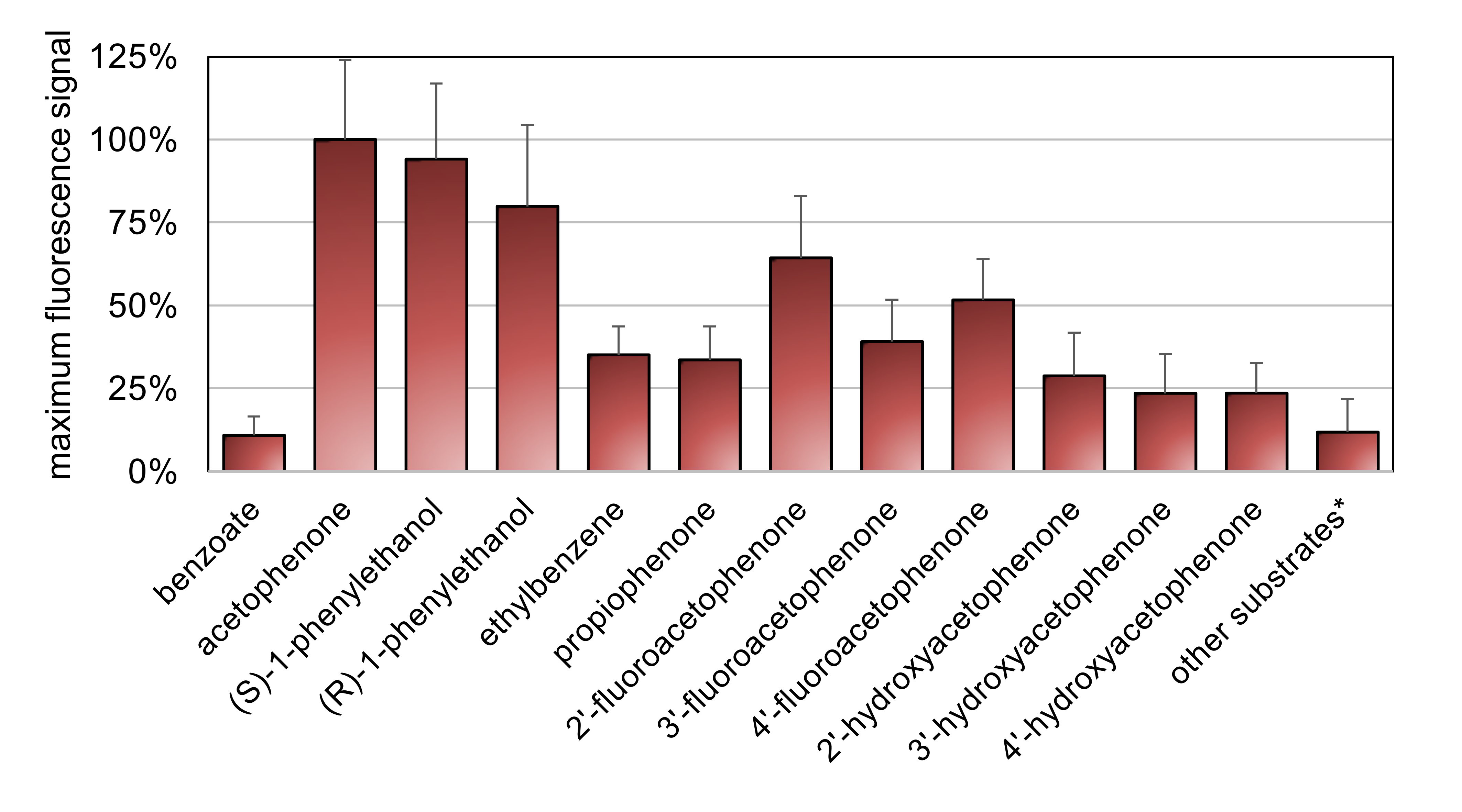

Supplement: Supplementary file 4 [file Image_3.TIF]

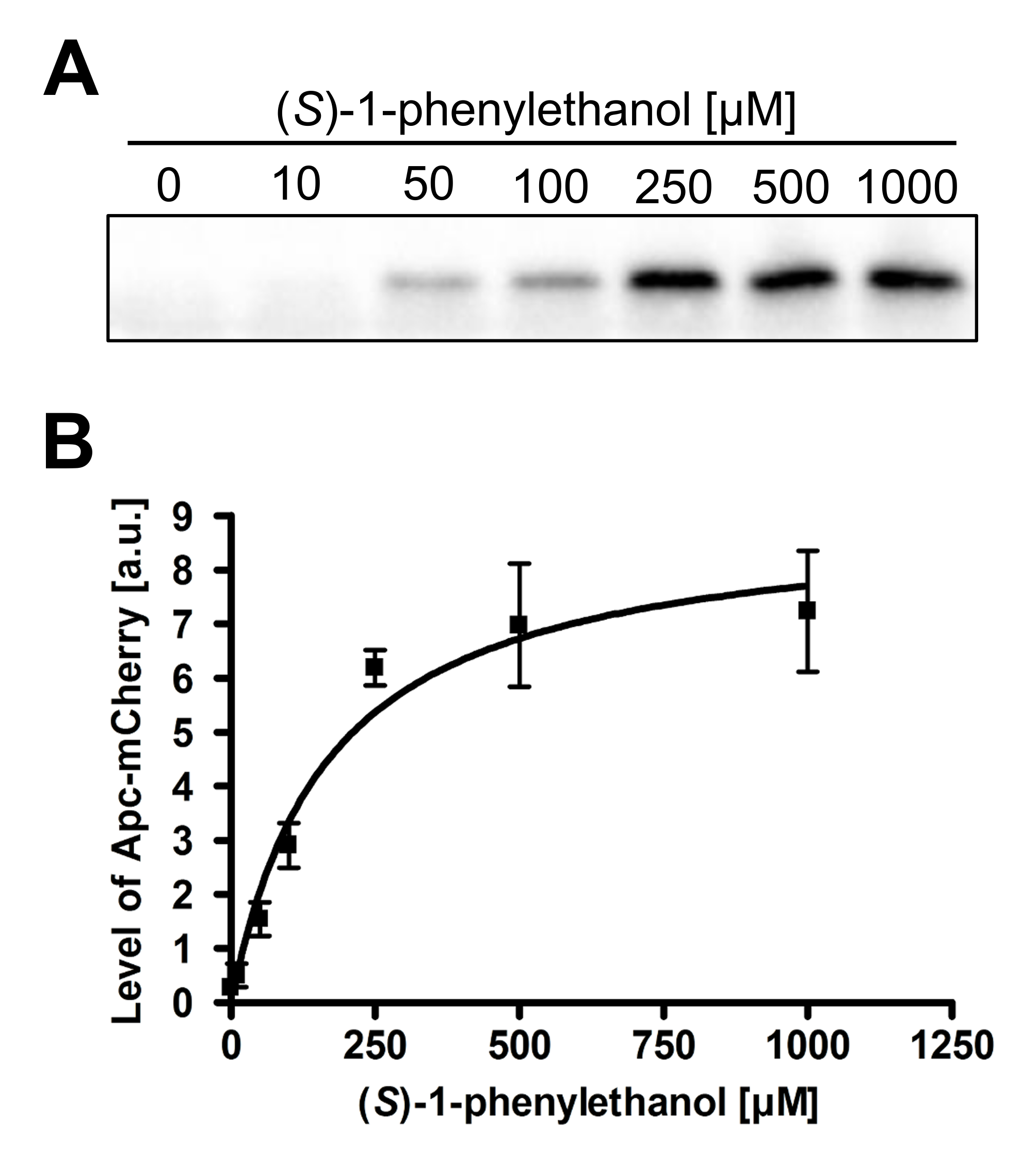

Supplement: Supplementary file 5 [file Image_4.TIF]

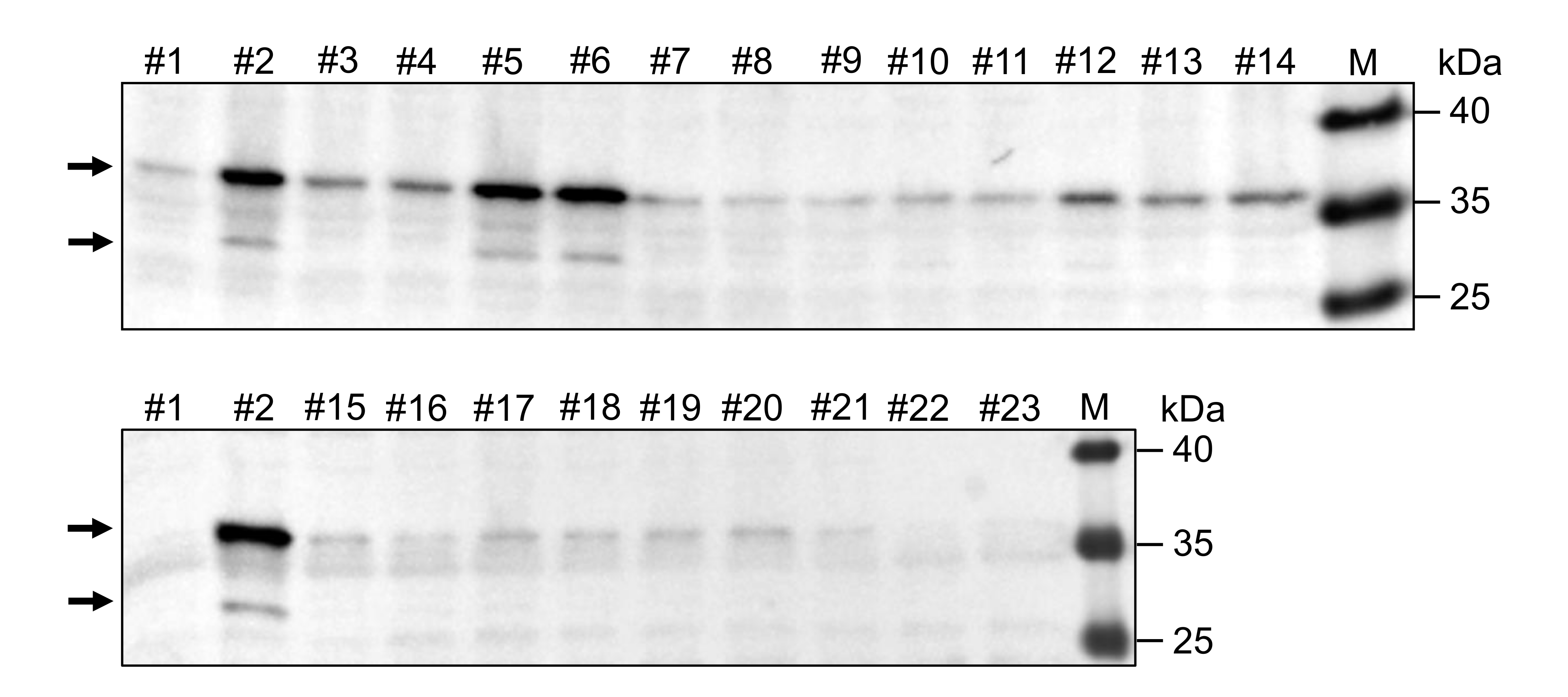

Supplement: Supplementary file 6 [file Image_5.TIF]
